# Clinical assessments and gait analysis for patients with Trimalleolar fractures in the early postoperative period

**DOI:** 10.1186/s12891-022-05615-z

**Published:** 2022-07-12

**Authors:** Ting Zhu, Ya Wang, Fei Tian, Wenjin Wang, Rongzhou Zhong, Hua Zhai, Shaobai Wang

**Affiliations:** 1 Key Laboratory of Exercise and Health Sciences of Ministry of Education, School of Kinesiology, Shanghai University of Sport, Research Building Room No. 412, Hengren Road No. 200, Shanghai, 200438 China; 2 Yangzhi Affiliated Rehabilitation Hospital of Tongji University (Shanghai Sunshine Rehabilitation Center), Building No.2, Guangxing Road No. 2209, Shanghai, 201619 China; 3 Department of Rehabilitation Medicine, Heping Hospital Affiliated to Changzhi Medical College, Changzhi, 046000 Shanxi China

**Keywords:** Ankle trimalleolar fracture, Clinical assessments, Biomechanics, Gait

## Abstract

**Background:**

Trimalleolar fracture is a common ankle fracture with serious complications and costly healthcare problem. Most studies used clinical assessments to evaluate the functional status of the patients. Although clinical assessments are valid, they are static and subjective. Dynamic, objective and precise evaluations such as gait analysis are needed. Ankle biomechanics studies on gait in patients with trimalleolar fractures are still rare. This study aimed to investigate the clinical outcomes and gait biomechanics in patients with trimalleolar fractures in the early postoperative period and compared to healthy controls.

**Methods:**

This was a cross-sectional study. 12 patients with trimalleolar fractures were recruited, and 12 healthy people served as controls. All patients underwent clinical assessments: Olerud and Molander ankle score (OMAS), ankle swelling and passive range of motion (ROM) of ankle, and completed gait biomechanical analysis when weight-bearing was allowed: temporal-spatial parameters, plantar pressure distributions, and surface electromyography (sEMG). The control group only performed gait test.

**Results:**

Patients had poor outcomes of clinical assessments in the short-term. During gait analysis, patients presented compromised gait patterns: shorter step length, larger step width, slower walking speed and shorter single support compared to healthy controls (*P* < 0.001), and patients showed asymmetrical gait. Symmetry index of step width and walking speed were mainly correlated with the difference of ankle inversion ROM between two sides (*R* = -0.750, *P* = 0.005; *R* = -0.700, *P* = 0.011). During walking, patients showed abnormal dynamic plantar pressure features (mainly in the hindfoot and forefoot regions), and the IEMG (integrated electromyography) of tibial anterior muscle (TA) and peroneal longus muscle (PL) were larger than healthy controls (*P* = 0.002, 0.050).

**Conclusions:**

Patients with trimalleolar fractures showed physical impairments of the ankle, and presented altered gait parameters compared to healthy subjects in the short-term. The ankle stability of patients declined, and deficits in TA and PL muscle ability might contribute to it. Restoring complete muscle functions and improving passive ankle ROM are significant to promote the recovery of a normal gait pattern.

## Introduction

Ankle fractures are one of the most common lower limb fractures [1]. Based on the number of malleoli involved, ankle fractures can be classified into isolated malleolar fractures, bimalleolar fractures and trimalleolar fractures, which occur in around 66, 25 and 7% of all ankle fractures respectively [2]. The trimalleolar fractures have worse clinical outcomes and an increased incidence of osteoarthrosis [2], and a notable proportion was unable to return to sports [3].

Although some studies investigated the differences among subgroups based on the severity of fractures [1, 4, 5], there were few studies focused on the characteristics of trimalleolar fractures alone [6]. Most studies used radiographic assessment, physical examination and patient-reported outcome measures (PROM) to evaluate the functional status of the patients post ankle fractures surgeries [1, 4]. Although these methods are considered to be valid, they are static and subjective, and dynamic, objective and precise evaluations are needed.

Gait is the most common and fundamental method for humans to perform physical activities, and the integrated actions of the nervous, muscular and skeletal systems are reflected in walking [7]. Gait analysis is a significant way of characterizing functional performance of humans, including temporal-spatial parameters (TSPs), gait kinematics, gait kinetics and musculoskeletal activity [8, 9]. TSPs are referred to as the vital signs of gait, including walking speed, cadence, step length and so on [8]. Plantar pressure distribution (PPD) includes many valuable information for evaluating stability and motor control ability of ankle, and it had been estimated in subjects who were at risk of sustaining ankle injuries or ankle instabilities [10]. The lower limb muscles are the active part of walking, and analyzing muscle activation during functional tasks, such as walking, would be more comprehensively to evaluate the ankle function [9]. Several studies have investigated lower extremity muscle activation by surface electromyography (sEMG) in patients with chronic ankle instability, ankle sprains and so on [7, 11]. Biomechanical studies on gait in patients with trimalleolar fractures are still rare, and to the best of our knowledge, this was the first study to exclusively explore the TSPs, PPD and sEMG characteristics of trimalleolar fractures patients.

The primary aim of this study was to investigate the gait biomechanics including TSPs, PPD and sEMG in patients with trimalleolar fractures during walking, and the results were compared with healthy controls. The secondary aim was to evaluate the association between gait parameters and ankle clinical assessments.

## Methods

### Participants

12 patients with unilateral trimalleolar fracture postoperatively were recruited as the experimental group and 12 healthy subjects as the control group. The inclusion criteria of the experimental group were as follows: age between 20 ~ 55 years, who were in the recovery period after the fixation of their trimalleolar ankle fractures, and were able to walk independently without the help of aids. Patients were excluded if they 1) isolated malleolar or bimalleolar fractures; 2) open fractures, pathological fractures, multiple injuries; 3) combined with injuries of blood vessels or nerves; or 4) unwilling to sign informed consent. Patients were recruited to the study during their follow-up examination in the Shanghai Sunshine Rehabilitation Center. All patients were diagnosed with a trimalleolar ankle fracture at the emergency department. Diagnosis was confirmed by clinical examination and a radiograph. A computed tomography scan was performed when necessary. All patients were treated operatively with open reduction and internal fixation following AO/ASIF principles within 2 weeks after injury. Patients who had a syndesmotic disruption were treated with a 3.5-mm cortical screw, which was removed 2 months post-surgery. Postoperatively, a plaster cast in neutral position was applied for 6 weeks. Patients were instructed to start toes and knee flexion and extension exercises immediately after surgery. Partial weight-bearing walking with crutches under the assistance from the physiotherapist was requested 6 weeks after surgery. Patients were approached at routine rehabilitation programs, including ankle passive mobilizations, muscle strength training, proprioception and balance training, and walking. Radiographic examinations including anteroposterior and lateral X-rays of the ankle taken at 3 and 6 months postoperatively. The control group required the subjects to be in good health, aged between 20 ~ 55 years, had no history of ankle joint injury or surgery, had not received ankle joint movement control training in the past year. Exclusion criteria were: 1) any hip, knee and ankle injuries affecting joint activity and diseases of the motor system; 2) abnormal lower limb alignment; 3) acute injury of the musculoskeletal structure of other joints within the previous 3 months; 4) patients with chronic ankle instability; 5) unwilling to sign informed consent. In this study, all healthy subjects were right leg dominant (the preferred leg to kick a soccer) [4]. All participants had detailed procedures introduced to them before performing experiment and signed the informed consent forms. This study was approved by the Medical Ethics Committee of Shanghai Sunshine Rehabilitation Center.

### Equipment

Walking performance were captured using the eight-cameras Vicon infrared motion capture system (200 Hz) (ViconT40, OxfordMetrics, Oxford, UK). Spherical reflective markers were placed on 21 specific anatomic points on the subjects: anterior superior iliac spine, posterior superior iliac spine, middle thigh, medial knee, lateral knee, middle leg, heel, second metatarsal, medial malleolus, lateral malleoluss on both sides of the body and midpoint of upper margin of sacrum. Vicon Nexus was used to process the 3D motions on the computer. The kinetic parameters were generated using the Vicon Plug-in-Gait model. Gait events were identified from force plates (Kistler Instrumentation Corp., Winterhur, Switzerland) data. The plantar pressure during walking was measured by an Emed® -× 400 plantar pressure system (100 Hz, 700 mm × 400 mm, 4 sensors/cm^2^.) (Novel GmbH, Munich, Germany). The sEMG signals were collected using a 16-bit Noraxon data acquisition system (1500 Hz) (Noraxon DTS, USA). According to SENIAM international standards, disposable Ag/AgCl circular bipolar electrodes were used (diameter:10 mm; inter-electrode spacing: 2 cm) [12].

### Testing procedures

After inclusion, the basic characteristics were obtained firstly. Then, patients underwent clinical examinations: assessments of the Olerud and Molander Ankle Score (OMAS), ankle circumference, and passive ankle joint mobility. Finally, the biomechanics of gait (temporal-spatial parameters, plantar pressure and sEMG) were tested.

### Clinical assessment

#### OMAS

OMAS is a PROM consisting of nine Likert-styled questions for symptom evaluation in patients with an ankle fracture. It was scored 0–100; with higher scores indicating better ankle function [13].

#### Ankle circumference

The circumferences of the ankles were measured with a flexible ruler without elasticity, wrapping around the ankle in a figure of eight [14]. Ankle swelling was quantified by comparing a patient’s injured and noninjured ankle girth difference.

#### Passive ankle joint mobility

Joint mobility test was conducted based on standard procedures. Range of motion (ROM) in the sagittal plane (dorsiflexion and plantarflexion) and the coronal plane (inversion and eversion) were measured with a manual goniometer. When measuring ROM, the patients were instructed to bring the ankle actively into maximum position, and then the ankle was passively brought to the maximum angle possible by a therapist [7, 15]. Dorsiflexion was measured in an extended knee position and with the ankle in the neutral position.

### Gait analysis

All participants underwent gait motion capture by Vicon. Participants were asked to walk barefoot at their preferred walking speed, and six walking trials were recorded. Before the formal testing of plantar pressure, patients were instructed to walk at a preferred walking speed along the walkway for 2 trails to become familiar with the procedures. Then five successful trials in barefoot walking were analysed. When measuring sEMG, electrodes were placed on tibial anterior muscle (TA), peroneal longus (PL), gastrocnemius medial (GM), gastrocnemius lateral (GL) and soleus muscle (S) of both sides. Then the maximal voluntary isometric contractions (MVIC) of these muscles were measured, which were used for normalizing sEMG signal. Two repetitions of every MVIC measurement were performed, with a two-minute rest period between test. Before walking testing, subjects performed a 5-minute walking warm-up at a self-selected pace. Participants completed a total of six walking trials, and sEMG data were collected for 30 seconds for each trail. Finally, filtering, full-wave rectified and smoothing were performed to process the data of MVIC and the EMG signal in walking. The maximal sEMG signal value was used for the normalization of the walking [16].

### Statistical analysis

Analyses were performed using SPSS Software (Version 23, Chicago, IL). All data was checked for normality through the Shapiro-Wilk test. Descriptive statistics were used to present the baseline characteristics of participants. Paired-sample t-tests were used to compare the gait analysis of the injured legs (*n* = 12) with the noninjured ankles (n = 12) of the patients. All gait parameters were compared between the injured legs (n = 12) of patients and with both legs (*n* = 24) of the healthy controls by 2- tailed, independent t tests. Some studies concluded that the right and left ankle/foot were independent, therefore, some authors suggested consideration of both limbs in the analysis [7, 17]. Finally, Pearson’s tests were performed to verify the correlation between the clinical assessments and temporal-spatial parameters of walking. Correlations were classified [direct (+) or inverse (−)] as weak (0–0.3), moderate (0.3–0.5), or strong (0.5–1) [7]. Differences were considered statistically significant at *P* values < 0.05.

## Results

### Participant characteristics

The demographic characteristics and OMAS for the participants were presented in Table 1. 12 patients and 12 healthy subjects were included in the trimalleolar fractures group and healthy control group respectively. There were no differences in baseline characteristics between the 2 groups. Three patients had ankle dislocation. One patient had syndesmosis damage. The injury causes included traffic accidents in four cases, fall from a height in four cases, simple fall injury in two cases, and the bruise injury caused by heavy object in two cases. Postoperative radiographs showed the fracture line became blurred and blunt, and foot joint space was clear. Gait analysis were performed at a mean of 4.50 ± 1.19 (range 3-7) months postoperatively. The mean OMAS score of patients was 56 ± 11.26 (range 35-75).

**Table 1:** Baseline characteristics of participants (Mean ± SD)

	Trimalleolar fractures (*n* = 12)	Healthy controls (n = 12)	*P* Value
Age (years)	42.20 ± 10.20	32.00 ± 13.98	0.070
Height (cm)	164.30 ± 7.45	165.83 ± 8.35	0.658
Weight (kg)	63.90 ± 8.67	61.75 ± 7.18	0.532
BMI (kg/m2)	23.60 ± 2.32	22.41 ± 1.52	0.159
Male/female	7/5	6/6	0.682
Injured/Dominant foot (n): right/left	8/4	12/0	
Time from surgery to gait analysis (mo)	4.50 ± 1.19 (3-7)		
OMAS, mean (range)			
Pain	21 ± 2.11 (20-25)		
Stiffness	3 ± 4.83 (0-10)		
Swelling	2 ± 2.58 (0-5)		
Stairs climbing	6 ± 2.11 (5-10)		
Running	0 ± 0 (0-0)		
Jumping	0 ± 0 (0-0)		
Squatting	3 ± 2.58 (0-5)		
Supporting	6.5 ± 4.74 (0-10)		
Work activity of daily life	14.5 ± 2.84 (10-20)		
Total score	56 ± 11.26 (35-75)		

*SD* Standard deviation, *BMI* Body mass index, *OMAS* The Olerud and Molander Ankle Score

### Clinical assessment: comparison between the injured and noninjured side

Circumference ankle was significantly larger on the injured side compared with the noninjured side in patients (*P* < 0.001), and the degree of ankle swelling was 1.41 ± 0.61 cm (Table 2). The passive ROM in dorsiflexion, plantar flexion, inversion, and eversion of the injured side were significantly smaller than those of the noninjured side (*P* = 0.002, 0.004, < 0.001, 0.004, Table 2).

**Table 2:** Clinical assessments of the patients with trimalleolar fractures (Mean ± SD)

	Injured side (n = 12)	Noninjured side (n = 12)	*P* value	Difference
Circumference ankle (cm)	50.73 ± 3.11	49.32 ± 2.98	<0.001	1.41 ± 0.61
ROM dorsiflexion (degree)	7.08 ± 3.91	13.08 ± 4.10	0.002	6.00 ± 5.13
ROM plantar flexion (degree)	33.33 ± 8.07	41.41 ± 7.83	0.004	8.08 ± 7.77
ROM inversion (degree)	13.75 ± 6.78	26.25 ± 9.64	<0.001	12.50 ± 7.76
ROM eversion (degree)	6.00 ± 4.17	13.25 ± 5.77	0.004	7.25 ± 6.92

*SD* Standard deviation, *ROM* Range of motion. The difference of ankle joint circumference is swelling. Swelling = (Circumference of injured side) - (Circumference of noninjured side), and the difference of angle = (ROM of noninjured side) - (ROM of injured side)

### Gait analysis: comparison between the injured and noninjured side and between patients and healthy subjects

#### Temporal-spatial parameters

The temporal-spatial gait parameters were as shown in Table 3. The standardized single-support time was expressed by the ratio of the single-support time to the total gait cycle to eliminate individual difference. The symmetry index is calculated using the formula: ((involved-uninvolved)/(involved+uninvolved)/2) × 100. An absolute value of symmetry index of zero indicates perfect symmetry and up to 5% difference between limbs is considered normal [1].

**Table 3:** Temporal-spatial gait parameters of the patients with trimalleolar fractures and both sides of the healthy controls

	Injured side (*n* = 12)	Noninjured side (n = 12)	Healthy controls (*n* = 24)	*P1 value*	*P2* value	*P3* value
Step length (m)	0.48 ± 0.13	0.41 ± 0.15	0.66 ± 0.06	<0.001	<0.001	0.008
Step width (m)	0.18 ± 0.04	0.18 ± 0.04	0.12 ± 0.03	<0.001	<0.001	0.434
Walking speed (m/s)	0.65 ± 0.29	0.66 ± 0.29	1.29 ± 0.13	<0.001	<0.001	0.041
Single support time (%)	16.27 ± 5.63	20.40 ± 2.71	32.27 ± 2.96	<0.001	<0.001	0.008
Symmetry index step length (%)	21.44 ± 26.34		−0.49 ± 6.02	0.014		
Symmetry index step Width (%)	−0.48 ± 7.07		−4.65 ± 14.17	0.397		
Symmetry index walking speed (%)	−3.74 ± 4.01		−0.35 ± 3.67	0.052		
Symmetry index single support time (%)	−27.63 ± 27.35		−0.94 ± 7.23	0.028		

*P1* Injured side VS healthy controls, *P2* Noninjured side VS healthy controls, *P3* Injured side VS noninjured side

Compared with the noninjured side, the step length, walking speed and single support time of the injured side in the patients were significantly decreased (*P* = 0.008, 0.041, 0.008, Table 3), while the step width was similar between both sides (*P* = 0.434). Compared with the healthy subjects, injured side and noninjured side in patients all demonstrated shorter step length (*P*<0.001), wider step width (*P*<0.001), slower walking speed (*P*<0.001) and less single support time (*P*<0.001). The symmetry indexes indicated asymmetry in patients and symmetry in healthy subjects. The symmetry index of the step length and single support time was significant different between the 2 groups (*P* = 0.014, 0.028), while the symmetry index of step width and walking speed was similar between the 2 groups (*P* = 0.397, 0.052).

#### Dynamic plantar pressure parameters

Compared to the noninjured side, the injured side demonstrated lower peak plantar pressure for the T345 (injured side: 61.09 ± 60.13 kpa, noninjured side: 112.27 ± 79.12 kpa, *P* = 0.022) and smaller contact area for the MF (injured side: 22.60 ± 5.64 cm^2^, noninjured side: 25.87 ± 7.90 cm^2^, *P* = 0.038). The peak plantar pressure, contact area and contact time in other regions were similar in both sides (*P* > 0.05).

As shown in Table 4, compared to the healthy group, the trimalleolar fractures group demonstrated lower peak pressure for the HF, M2, M3, T2, T345 (*P* = 0.040, <0.001,<0.001, 0.001, 0.003). The contact areas of the HF, M4, T1, T2, T345 in the trimalleolar fractures group were smaller (*P*<0.001, 0.017, 0.004, 0.002,<0.001). The contact time for the HF and MF in the trimalleolar fractures group was increased (*P*<0.001, 0.001), and the contact time for the T2, T345 was reduced (*P* = 0.001,0.027). The total contact time of the patients and healthy group were (981.00 ± 141.45) ms and (791.14 ± 111.05) ms respectively, *P* = 0.003.

**Table 4:** Comparison of dynamic plantar pressure features between the injured sides of patients with trimalleolar fractures and both sides of the healthy controls

Region	Peak plantar pressure (kpa)		Contact area (cm^2^)		Contact time (%)	
	Trimalleolar fractures (*n* = 12)	Healthy controls (n = 24)	Trimalleolar fractures (n = 12)	Healthy controls (n = 24)	Trimalleolar fractures (n = 12)	Healthy controls (n = 24)
HF	290.95 ± 54.45*	358.12 ± 96.90	28.48 ± 3.07*	30.78 ± 3.59	71.06 ± 7.18*	57.33 ± 7.17
MF	127.68 ± 39.75	155.37 ± 47.08	22.60 ± 5.64	25.81 ± 5.32	72.18 ± 7.22*	66.10 ± 5.57
M1	253.00 ± 138.61	277.83 ± 101.60	11.20 ± 2.24	11.93 ± 1.20	79.97 ± 9.04	82.16 ± 3.76
M2	226.22 ± 144.40*	597.62 ± 292.83	9.05 ± 1.23	9.31 ± 0.77	85.32 ± 6.57	84.62 ± 3.61
M3	245.45 ± 147.56*	455.67 ± 149.56	9.97 ± 1.40	10.69 ± 0.92	86.76 ± 6.68	85.55 ± 3.11
M4	192.50 ± 113.27	246.37 ± 39.19	7.97 ± 1.04*	8.75 ± 0.74	83.40 ± 6.60	83.71 ± 3.31
M5	125.22 ± 72.11	184.00 ± 96.74	5.03 ± 0.58	5.43 ± 0.68	76.60 ± 6.32	75.96 ± 4.67
T1	300.18 ± 286.80	417.42 ± 141.87	7.45 ± 2.31*	10.04 ± 0.99	75.26 ± 20.24	73.35 ± 11.52
T2	79.27 ± 81.91*	211.75 ± 104.20	2.25 ± 1.37*	3.92 ± 0.54	43.78 ± 20.71*	65.22 ± 13.57
T345	61.09 ± 60.13*	148.08 ± 78.35	2.81 ± 2.17*	6.84 ± 2.03	42.57 ± 26.22*	63.40 ± 12.06

*HF* Hindfoot, *MF* Medial midfoot, *M1* The first metatarsal head, *M2* The second metatarsal head, *M3* The third metatarsal head, *M4* The fourth metatarsal head, *M5* The fifth metatarsal head, *T1* Hallux, *T1* The second toe, *T3-5* The third to fifth toes

*P < 0.05

#### sEMG

The integrated EMG (%) (IEMG) of TA of the injured side (10.84 ± 4.59) in the patients was significantly larger than the noninjured side (8.93 ± 4.56), *P* = 0.014. While the IEMG (%) of PL (injured side: 29.51 ± 20.43, noninjured side: 54.35 ± 57.27, *P* = 0.117), GL (injured side: 27.91 ± 17.78, noninjured side: 32.13 ± 19.75, *P* = 0.581), GM (injured side: 24.78 ± 17.83, noninjured side: 34.44 ± 22.77, *P* = 0.124) and S (injured side: 21.30 ± 12.65, noninjured side: 21.94 ± 14.41, *P* = 0.922) were not significant different between both sides.

**Fig. 1:**
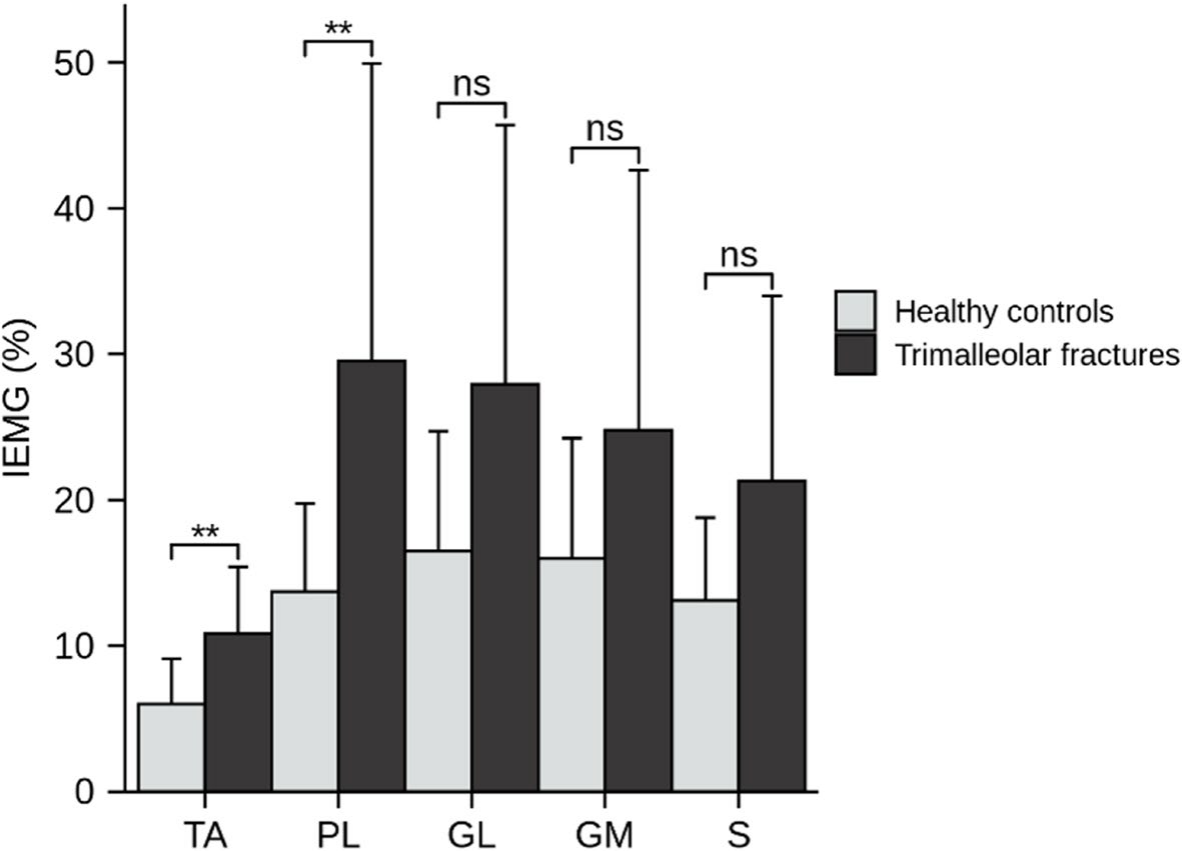
Comparison of electromyography characteristics between the injured sides of patients with trimalleolar fractures and both sides of the healthy controls. TA: tibial anterior muscle, PL: peroneal longus, GM: gastrocnemius medial, GL: gastrocnemius lateral, S: soleus muscle. IEMG: integrated electromyography. ** *P* < 0.05. ns: no significant difference

During walking, the IEMG (%) of TA, PL were significantly higher in the patients (*P* = 0.002, 0.008) than in the healthy controls, while no differences were identified between groups for IEMG (%) of GL, GM and S (Fig. 1).

#### Correlations analysis

**Fig. 2:**
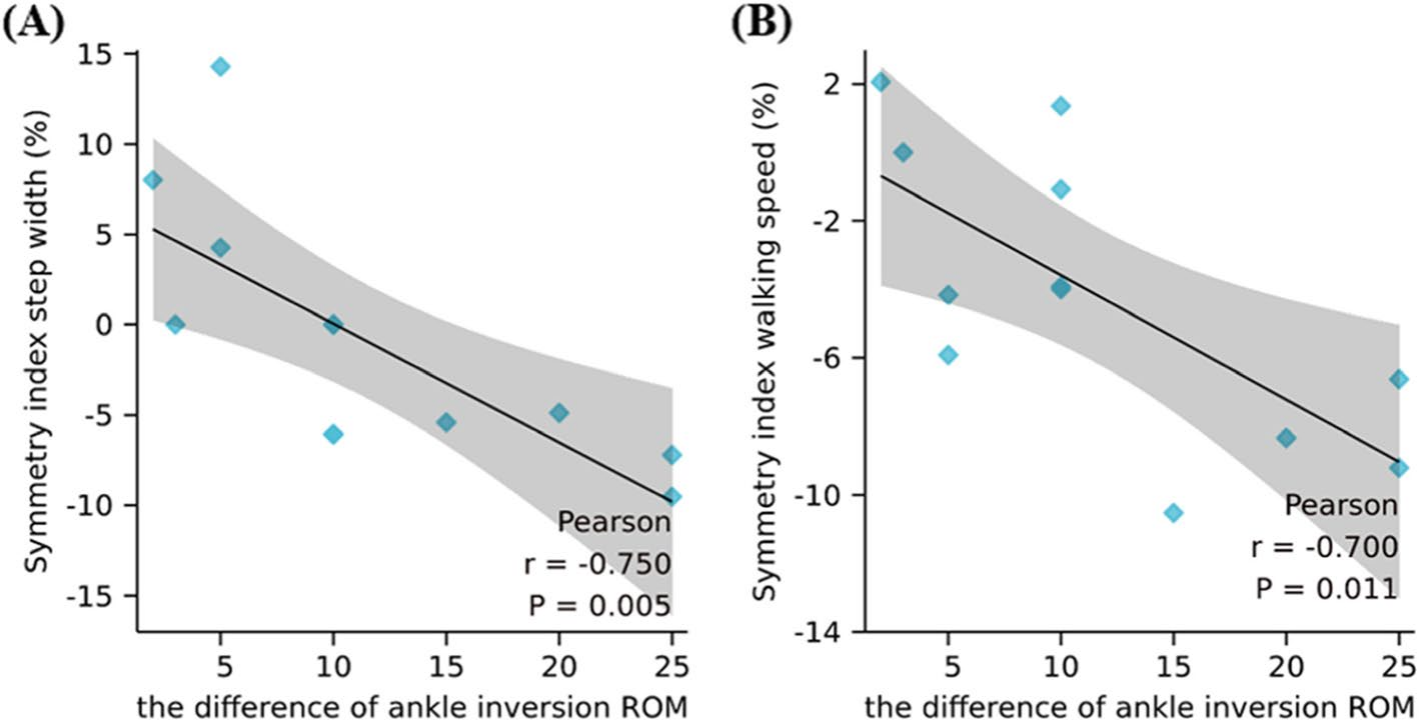
Correlations between the symmetry index (**A**) step width, (**B**) walking speed) and the difference of ankle inversion ROM. ROM: range of motion

The symmetry index of step width and walking speed were highly inversely correlated with the difference of ankle inversion ROM between two sides (*R* = -0.750, *P* = 0.005, Fig. 2A; *R* = -0.700, *P* = 0.011, Fig. 2B). The symmetry indexes were not correlated with other clinical assessments of the ankle (*P* > 0.050).

## Discussion

This study indicated that at an average of 4.5-months post-surgery, patients with trimalleolar fractures showed poor OMAS results, and their injured ankles were swollen, and the passive ROM were decreased. During gait analysis, patients demonstrated abnormal gait compared with healthy controls, and an asymmetrical gait pattern was seen in patients. Compared with healthy controls, the abnormal performance of patients in plantar pressure distribution were concentrated in hindfoot and forefoot, and patients also showed abnormal muscle activity of TA and PL. Furthermore, the passive inversion ROM was highly correlated to symmetry index of step width and walking. This study was the first to indicate remaining detailed gait deficits in patients with trimalleolar fractures. In addition, gait parameters were correlated with clinical outcomes in patients with trimalleolar fractures for the first time.

OMAS are usually used as a reliable and valid outcome measure after an ankle fracture [18], and based on the total score, ankle function of patients could be divided into four grades: excellent (OMAS:100 to 91 points), good (OMAS:90 to 61 points), fair (OMAS:60 to 31 points) and poor (OMAS:30 to 0 points) [19]. In this study, the mean OMAS was 56, indicating that patients with trimalleolar fractures reported fair ankle function. According to the study, the disability of running and jumping contributed most to the total score. Several studies also investigated the OMAS of ankle fractures patients, and they showed better results than our study. Oguzhan Tano glu et al. [20] compared the effect of a 1-stage surgery for the unstable malleolar fracture dislocations with the 2-stage surgery. The two group all included patients with isolated malleolar fractures, bimalleolar fractures and trimalleolar fractures. And the duration of follow-up of the two group was 21.7 and 19.2 months respectively. The mean OMAS was 87.8 for the 1-stage surgery group and 83.2 for the 2-stage surgery. Mareen Braunstein et al. [21] demonstrated functional outcomes after 1 year of arthroscopically assisted ankle fracture treatment, and they reported a mean OMAS of 85 for trimalleolar fractures. The poor OMAS results for trimalleolar fractures in this study might be mainly attributed to the short length of the postoperative follow-up period. This study investigated the clinical outcomes after 4.5 months postoperatively, while other studies evaluated the long-term (more than 1 year) clinical outcomes.

This study indicated that patients with trimalleolar fractures remained physical impairments postoperatively. Compared with the noninjured side, patients represented ankle swelling, and a decrease in passive ROM on the injured side. Ankle swelling is a common and long-standing complication after surgery. It was reported that more than half of the patients following unimalleolar and bimalleolar ankle fractures presented stiffness, swelling and pain [1]. 60% or more of the patients 65 years or older reported ankle pain, swelling and problems when using stairs and reduced activities of daily life 1 year after ankle fractures [22]. Shah et al .[23] demonstrated that around 45% of 69 patients with Weber B and C ankle fractures still had ankle swelling at 5 years after the injury. Our study investigated the passive ROM, and mean (sd) angle of dorsiflexion, plantarflexion, inversion and eversion of the injured leg on the trimalleolar fractures patients was 7.08 (3.91) degrees, 33.33 (8.07) degrees, 13.75 (6.78) degrees and 6.00 (4.17) degrees respectively. Ganit Segal et al. [1] also measured the passive ankle ROM of patients with trimalleolar fractures in the sagittal (dorsiflexion/ plantar flexion) and coronal plane (inversion/eversion), and the ROM was − 0.8 (7.6) degrees, 40.6 (7.5) degrees, 5.6 (3.6) degrees and 2.5 (4.0) degrees respectively. The result was a little different from our study, might due to the assessment point. Ganit Segal et al. [1] measured ROM at 64.5 days from injury, which was earlier than ours (4.5 months). There was also a study evaluated ROM during activities. Van Hoeve, S et al. [4] found that compared with the healthy subjects (12.59 ± 3.73 ), the ROM during gait in patients (7.13 ± 2.55 ) with trimalleolar ankle fractures decreased significantly. After ankle fractures, the uncoagulated hemorrhage leads to the rapid increase of intra-articular pressure, which causes abrupt joint swelling, pain and limited mobility [24]. Presence of soft tissue damages such as tendon and ligamental injuries can cause chronic swelling and stiffness therefore resulting in the dismal outcome [3]. These complications might alter the gait.

The present study showed that patients with trimalleolar fractures presented compromised gait pattern. The temporal-spatial parameters of the injured side and noninjured side were significantly different from healthy subjects, and the two sides were also different, except no difference exist in step width. This is in line with other research findings. Three studies investigated the gait parameters of patients with trimalleolar fractures, and compared those with healthy group. They found all gait parameters were significantly below the normal range [1, 4, 6]. Andrew F. Tyler et al. [6] also showed that the gait characteristics of ankle fracture patients were more similar to healthy elderly patients. However, these studies only investigated the differences between patients and healthy subjects, but did not compare the gait parameters of the injured side with the noninjured side. Ganit Segal et al. [1] also examined limb symmetry of the gait patterns, and found significant asymmetry in step length and single limb support. These findings were consistent with this study. And by correlation analysis in this study, step asymmetry might be related to the difference of ankle inversion ROM between two sides. All these results presented that patients with trimalleolar fractures did not achieve restoration of normal physiologic gait in the short-term, and patients adopted a simple security strategy with a reduction of walking speed [7]. In addition, many studies indicate that gait changes over an individual’s lifetime [25, 26]. With aging, there are physiological changes in the sensorimotor systems, which may cause gait abnormalities. In this study, the age span for each group is relatively large, this may be a bias factor contributing to the abnormal gait features. The results should be further confirmed by shrinking the age span.

The differences of features of plantar pressure distributions and sEMG in both sides also indicated asymmetries in gait in patients with trimalleolar fractures. The plantar pressure in the T345 and the contact area of MF of the injured side were significantly smaller than those of the noninjured side, and this was probably due to a more cautious and compensatory walking pattern, by further biasing the center of gravity to the noninjured side. Sjoerd Kolk et al .[27] also showed subtle asymmetries in gait kinetics and kinematics between the operated and non-operated limbs, and they considered that patients performed a more cautious walking pattern and an integral strategy. Plantar pressures of other type ankle fractures such as pilon fractures, calcaneal fractures were also asymmetry, and adhesion or conduction disorders at the tibia may be causes of abnormal plantar pressure [28, 29].

Compared with normal healthy subjects, patients with trimalleolar fracture performed abnormal gait during walking support period. Patients tended to step more cautiously on the injured heel, showing smaller peak plantar pressure in HF, and this might be due to pain or psychological factors such as fear or worry of reinjury [29]. For patients, the contact time (%) of HF and MF and total contact time were significantly increased, and it might be associated with lower ankle stability: for patients with trimalleolar fractures, the lateral, medial and posterior malleolus were injured, probably impairing the ankle stability, and patients needed more time to maintain ankle stability [1]. The muscle activity on the TA and PL of the injured side was significantly larger than those in the noninjured, also indicating the ankle stability of the injured side decreased, because muscular co-contraction of TA and PL was increased to stabilize the ankle joint [30]. And in this study, the step width was significantly smaller than that of healthy controls, also showing the walk stability decreased in patients. The plantar pressure features (smaller peak plantar pressure and contact area, shorter contact time) in the forefoot demonstrated that the propulsion ability during walking significantly decreased in patient, and this could decrease the walking speed. In this study, the IEMG of PL of patients was significantly increased, indicating the muscle ability decreased. Therefore, it was reasonable to speculate that the abnormal gait features in the forefoot might be related to the decreased ability of PL. During normal walking, the plantar pressure in the first metatarsal head plays a key role in pushing off [31]. And the PL is essential for maintaining ankle stability and plays an important role in the push-off stage. The PL origins at the proximal tibia and fibula, and inserts at the first metatarsal and medial cuneiform [32]. Except for contributing to 63% of eversion strength, the PL is helpful to initiate pronation and stabilize first ray during propulsion phases of gait [32].

Many studies indicated that speed has significantly influence on gait kinematics, kinetics, and sEMG. Sander van Hoeve et al. [4] found that when asked to walk at preferred normal speed, patients after ankle fractures showed lower walking speed compared to healthy subjects. When healthy subjects walked at equal speed, significantly lower ROM in the ankle flexion/extension during loading and push-off phases was also found in patients. And the smallest ankle flexion/extension ROM was found for the patients with trimalleolar fractures. Rachel M. Koldenhoven et al. [33] analyzed differences in gait characteristics between individuals with chronic ankle instability and healthy controls at three different walking speed (Preferred walking speed (PWS), 120% PWS, and standardized walking speed of 1.34 m/s). It found that the increases in walking speed magnified the differences between the groups for ankle inversion and hip adduction kinematics. But no group differences were identified for EMG variables, which might be due to the relatively small sample size and naturally high variability when measuring EMG activity. Gordon L. Warren [34] assessed the effects of walking speed (walked on a treadmill at seven speeds between 0.45 and 1.79 m/) on plantar pressures and lower-leg muscle activities in healthy participants. Except for the medial midfoot (*P* = 0.33), there were significant effects of speed on the peak values in the pressure–time curves for all plantar regions. There was also a highly significant effect of speed on the peak values in the root mean square –time curves for both the TA and GM muscles (*P* < 0.001). Preferred walking speed [33] is an individualized component of gait and should be considered when analyzing gait in a laboratory setting. However, standardizing walking speeds when measuring differences between groups is also important, which could improve the reproducibility of results between studies. Therefore, it is reasonable to speculate that walking speed may also affect gait parameters between trimalleolar fractures patients and healthy controls. More work needs to be done in this area to better assess gait performance in patients with trimalleolar fractures.

### Limitations

There were some limitations in this study. First, no age-matched control group was tested. The current healthy control group trended toward younger age than the fracture patients. This may be a bias factor contributing to the features noted in the gait analysis and may not be attributable to trimalleolar fractures. And the relatively small sample size may not be representative of the population of trimalleolar ankle fractures. Future work with a larger sample size and smaller age range will be required to fully investigate these characteristics. Second, this study did not divide the patients into “unfixed posterior malleoli group” and “fixed Posterior malleoli group”. However, it was demonstrated that fixation of the posterior malleolus particularly did not appear to improve gait characteristics. Therefore, the patients in this study were not further grouped into two subgroups. Third, the participants in this study were asked to walk at their natural speed, and these values in healthy subjects were found to be faster when compared to the patients, which may affect the comparisons between the other gait biomechanics parameters. Future research should examine the difference in gait patterns when patients walk at the same speed.

## Conclusion

Patients with trimalleolar fractures showed poor PROM results, ankle swelling and smaller passive ROM in the early postoperative period. Compared to healthy controls, patients showed altered temporal-spatial, plantar pressure and sEMG parameters. Gait asymmetries were correlated to the difference of ankle inversion ROM between two sides, and improving passive ankle ROM, particularly ankle inversion ROM, is significant to guarantee the recovery of gait symmetries. Restoring of TA and PL muscle functions should be progressively performed in the early postoperative period to increase the ankle stability and the propulsion ability of patients during walking.

## Data Availability

All data generated or analysed during this study are included in this published article.

## References

[1] Segal G, Elbaz A, Parsi A, Heller Z, Palmanovich E, Nyska M (2014). Clinical outcomes following ankle fracture: a cross-sectional observational study. J Foot Ankle Res.

[2] Testa G, Ganci M, Amico M, Papotto G, Giardina SMC, Sessa G (2019). Negative prognostic factors in surgical treatment for trimalleolar fractures. European J Orthop Surg Traumatol: orthopedie traumatology.

[3] Hong CC, Nashi N, Prosad Roy S, Tan KJ (2014). Impact of trimalleolar ankle fractures: how do patients fare post-operatively?. Foot Ankle Surg.

[4] van Hoeve S, Houben M, Verbruggen J, Willems P, Meijer K, Poeze M (2019). Gait analysis related to functional outcome in patients operated for ankle fractures, journal of orthopaedic research : official publication of the Orthopaedic research. Society..

[5] Wang R, Thur CK, Gutierrez-Farewik EM, Wretenberg P, Broström E (2010). One year follow-up after operative ankle fractures: a prospective gait analysis study with a multi-segment foot model. Gait Posture.

[6] Tyler A, Rose T, Day S, Kenia J, Horan A, Mehta S (2020). Comparison of spatiotemporal gait parameters following operative treatment of Trimalleolar ankle fractures vs healthy controls. Foot Ankle Orthop.

[7] Punt IM, Ziltener J-L, Laidet M, Armand S, Allet L (2015). Gait and physical impairments in patients with acute ankle sprains who did not receive physical therapy. PM&R.

[8] Kane JM, Coleman S, Brodsky JW (2017). Kinematics and function of Total ankle replacements versus Normal ankles. Foot Ankle Clin.

[9] Blair S, Lake MJ, Ding R, Sterzing T (2018). Magnitude and variability of gait characteristics when walking on an irregular surface at different speeds. Hum Mov Sci.

[10] Buldt AK, Forghany S, Landorf KB, Levinger P, Murley GS, Menz HB (2018). Foot posture is associated with plantar pressure during gait: a comparison of normal, planus and cavus feet. Gait Posture.

[11] Feger MA, Donovan L, Hart JM, Hertel J (2015). Lower extremity muscle activation in patients with or without chronic ankle instability during walking. J Athl Train.

[12] Campanini I, Disselhorst-Klug C, Rymer WZ, Merletti R (2020). Surface EMG in clinical assessment and neurorehabilitation: barriers limiting its use. Front Neurol.

[13] Olerud C, Molander H (1984). A scoring scale for symptom evaluation after ankle fracture, archives of orthopaedic and traumatic surgery. Archiv fur orthopadische und Unfall-Chirurgie.

[14] Pugia ML, Middel CJ, Seward SW, Pollock JL, Hall RC, Lowe L (2001). Comparison of acute swelling and function in subjects with lateral ankle injury. J Orthop Sports Phys Ther.

[15] Elveru RA, Rothstein JM, Lamb RL (1988). Goniometric reliability in a clinical setting. Subtalar and ankle joint measurements, Physical therapy.

[16] Bavdek R, Zdolšek A, Strojnik V (2018). Peroneal muscle activity during different types of walking [J]. J Foot Ankle Res.

[17] Aleixo P, Vaz Patto J, Cardoso A, Moreira H, Abrantes J (2019). Ankle kinematics and kinetics during gait in healthy and rheumatoid arthritis post-menopausal women. Somatosens Mot Res.

[18] Garratt AM, Naumann MG, Sigurdsen U, Utvåg SE, Stavem K (2018). Evaluation of three patient reported outcome measures following operative fixation of closed ankle fractures. BMC Musculoskelet Disord.

[19] Görtz S, De Young AJ, Bugbee WD (2010). Fresh osteochondral allografting for osteochondral lesions of the talus. Foot Ankle Int.

[20] Tanoğlu O, Gökgöz MB, Özmeriç A, Alemdaroğlu KB (2019). Two-stage surgery for the malleolar fracture-dislocation with severe soft tissue injuries does not affect the functional results. J Foot Ankle Surg.

[21] Braunstein M, Baumbach SF, Urresti-Gundlach M, Borgmann L, Böcker W, Polzer H (2020). Arthroscopically assisted treatment of complex ankle fractures: intra-articular findings and 1-year follow-up. J Foot Ankle Surg.

[22] Nilsson G, Jonsson K, Ekdahl C, Eneroth M (2007). Outcome and quality of life after surgically treated ankle fractures in patients 65 years or older. BMC Musculoskelet Disord.

[23] Shah NH, Sundaram RO, Velusamy A, Braithwaite IJ (2007). Five-year functional outcome analysis of ankle fracture fixation. Injury..

[24] Liu C, Guo J, Cui Q, Li D, Zeng Y (2016). Clinical and imaging analysis of subclinical hemophilia combined with coxarthrosis: case report and literature review. Springerplus..

[25] Beauchet O, Allali G, Sekhon H (2017). Guidelines for assessment of gait and reference values for spatiotemporal gait parameters in older adults: the Biomathics and Canadian gait consortiums initiative [J]. Front Hum Neurosci.

[26] Montero-Odasso M, Verghese J, Beauchet O (2012). Gait and cognition: a complementary approach to understanding brain function and the risk of falling [J]. J Am Geriatr Soc.

[27] Kolk S, Fluit R, Luijten J, Heesterbeek PJ, Geurts AC, Verdonschot N (2015). Gait and lower limb muscle strength in women after triple innominate osteotomy. BMC Musculoskelet Disord.

[28] van Hoeve S, de Vos J, Verbruggen JP, Willems P, Meijer K, Poeze M (2015). Gait analysis and functional outcome after calcaneal fracture, the journal of bone and joint surgery. American volume.

[29] Jandova S, Pazour J, Janura M (2019). Comparison of plantar pressure distribution during walking after two different surgical treatments for calcaneal fracture. J Foot Ankle Surg.

[30] Sasaki K, Neptune RR (2006). Differences in muscle function during walking and running at the same speed. J Biomech.

[31] Pu F, Ren W, Fu H, Zheng X, Yang M, Jan YK (2018). Plantar blood flow response to accumulated pressure stimulus in diabetic people with different peak plantar pressure: a non-randomized clinical trial. Med Biol Eng Comput.

[32] Koh D, Liow L, Cheah J, Koo K (2019). Peroneus longus tendon rupture: A case report. World J Orthop.

[33] Koldenhoven RM, Hart J, Saliba S (2019). Gait kinematics & kinetics at three walking speeds in individuals with chronic ankle instability and ankle sprain copers [J]. Gait Posture.

[34] Warren GL, Maher RM, Higbie EJ (2004). Temporal patterns of plantar pressures and lower-leg muscle activity during walking: effect of speed [J]. Gait Posture.

